# 3D Volumetric Mechanosensation of MCF7 Breast Cancer Spheroids in a Linear Stiffness Gradient GelAGE

**DOI:** 10.1002/adhm.202301506

**Published:** 2023-09-19

**Authors:** Danielle Vahala, Sebastian E. Amos, Marta Sacchi, Bram G. Soliman, Matt S. Hepburn, Alireza Mowla, Jiayue Li, Ji Hoon Jeong, Chrissie Astell, Yongsung Hwang, Brendan F. Kennedy, Khoon S. Lim, Yu Suk Choi

**Affiliations:** ^1^ School of Human Sciences The University of Western Australia Perth WA 6009 Australia; ^2^ Department of Orthopaedic Surgery and Musculoskeletal Medicine University of Otago Christchurch Christchurch 8140 New Zealand; ^3^ Department of Electrical Electronic & Computer Engineering School of Engineering The University of Western Australia Perth WA 6009 Australia; ^4^ BRITElab Harry Perkins Institute of Medical Research QEII Medical Centre Nedlands and Centre for Medical Research The University of Western Australia Perth WA 6009 Australia; ^5^ Soonchunhyang Institute of Medi‐Bio Science Soonchunhyang University Cheonan‐si Chungcheongnam‐do 31151 South Korea; ^6^ School of Medical Sciences University of Sydney Sydney NSW 2006 Australia

**Keywords:** breast cancer cell, cell volume, mechanotransduction, organoids, Rigidity, TRPV4

## Abstract

The tumor microenvironment presents spatiotemporal shifts in biomechanical properties with cancer progression. Hydrogel biomaterials like GelAGE offer the stiffness tuneability to recapitulate dynamic changes in tumor tissues by altering photo‐energy exposures. Here, a tuneable hydrogel with spatiotemporal control of stiffness and mesh‐network is developed. The volume of MCF7 spheroids encapsulated in a linear stiffness gradient demonstrates an inverse relationship with stiffness (*p* < 0.0001). As spheroids are exposed to increased crosslinking (stiffer) and greater mechanical confinement, spheroid stiffness increases. Protein expression (TRPV4, β1 integrin, E‐cadherin, and F‐actin) decreases with increasing stiffness while showing strong correlations to spheroid volume (*r* ^2^ > 0.9). To further investigate the role of volume, MCF7 spheroids are grown in a soft matrix for 5 days prior to a second polymerisation which presents a stiffness gradient to equally expanded spheroids. Despite being exposed to variable stiffness, these spheroids show even protein expression, confirming volume as a key regulator. Overall, this work showcases the versatility of GelAGE and demonstrates volume expansion as a key regulator of 3D mechanosensation in MCF7 breast cancer spheroids. This platform has the potential to further investigation into the role of stiffness and dimensionality in 3D spheroid culture for other types of cancers and diseases.

## Graphical abstract

There is a constant need for biomaterials that offer a high degree of tuneability to greater recapitulate the variable tissue properties noted in diseased tissues, such as breast cancer. Using a linear stiffness gradient GelAGE hydrogel, an increased density of mesh‐network results in restricted volume expansion of MCF7 spheroids and reduces key protein expression (TRPV4, β1 integrin, E‐cadherin, and F‐actin).

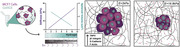

## 1 Introduction

The biochemical composition and biomechanical properties of the extracellular matrix (ECM) are extremely heterogenous and spatiotemporally dynamic throughout development,^[^ ^1^ ^]^ ageing,^[^ ^2^ ^]^ and disease.^[^ ^3^ ^]^ It is well known that extensive changes to the tumor microenvironment accompany cancer progression.^[^ ^4^ ^]^ In clinics, increased breast tissue density is asserted as a strong risk factor for breast cancer diagnosis.^[^ ^5^ ^]^ At a cellular level, stiffening of the ECM can result in elevated cytoskeletal tension due to altered focal adhesions leading to disrupted cell polarity and induction of a malignant phenotype in a stiffness‐dependent manner.^[^ ^5^, ^6^ ^]^

To recapitulate the complex properties of the tumor microenvironment (TME), biomaterials are continuously advancing to enable the tuneability of variable tissue properties and permit further investigation into cell–matrix interactions. Many studies still utilize 2‐dimensional and static biomaterials, such as polyacrylamide hydrogels, which have been critical in forming our understanding of stiffness‐dependent mechanisms including tissue stiffness‐specific stem cell differentiation.^[^ ^7^ ^]^ With enabling biomaterials, the investigations into 3‐dimensional cell–matrix interaction have allowed new understandings in 3D cell mechanosensation. These early investigations have examined mechanomarker expression (e.g., YAP nuclear translocalization and Lamin A/C expression) and unexpectedly shown an inverse trend in 3D when compared to 2D.^[^ ^8^ ^]^ This new data has prompted further research to investigate the role of dimensionality.

Photocrosslinkable hydrogels, such as gelatin methacryloyl (GelMA) and allylated gelatin (GelAGE) hydrogels, have gained significant traction in biomaterials due to their high tuneability and cytocompatibility.^[^ ^7^, ^9^ ^]^ As an alternative to the chain‐growth polymerization of GelMA, GelAGE utilizes step‐growth thiol‐ene click chemistry to facilitate crosslinking which yields more homogenous networks and subsequently maintains more stable matrices at lower stiffnesses.^[^ ^9a^ ^]^ Due to their photocrosslinkable nature, these hydrogels can be controlled spatially via a transparency gradient photomask which attenuates light differentially to produce a linear stiffness gradient. Gradient hydrogels that cover physiological stiffness ranges have enabled the generation of high throughput data which allows holistic understandings of mechanotransduction of stem cells and glioblastomas.^[^ ^8^, ^10^ ^]^ By utilizing linear stiffness gradient GelAGE hydrogels this study has investigated the mechanosensation of non‐metastatic breast cancer (MCF7) using a spheroid growth model within a pathophysiological stiffness range.

The overall mechanical properties of hydrogels are often associated with the degree of crosslinking. A stiffer hydrogel will have a higher degree of crosslinking, subsequently producing a tighter hydrogel network with a smaller mesh size. This stiffness—mesh size coupling can complicate the interpretation of mechanically induced cell fate.^[^ ^8d,e^ ^]^ Cell spreading (volume in 3D) has shown to have an inverse relationship to stiffness as a high level of crosslinking (stiffer) results in greater mechanical confinement.^[^ ^8^, ^11^ ^]^ To reduce the effects of mechanical confinement, ultimately leading to cell volume restriction, we developed a platform that utilizes two‐polymerization steps to control spheroid volume expansion independent of surrounding matrix stiffness. The first matrix with soft mechanics and large mesh‐network, relative to the stiff condition, reduced the cell volume restriction for the first 5 days. The already polymerized GelAGE with MCF7 cells was further stiffened to a linear stiffness gradient using a transparency photomask to expose MCF7 spheroids to variable stiffness for a further 5 days. This 2‐step polymerization platform allowed a segregated approach to interpreting MCF7 spheroid phenotype relative to volume and matrix stiffness.

Overall, this study showcases the possibility of recapitulating spatiotemporal dynamics in “in vivo” conditions, such as the breast tumor microenvironment, using a highly tuneable hydrogel platform. This platform has enabled investigation into the role of dimensionality in 3D mechanotransduction and has shown promising potential in experiments mimicking mechanical changes in other diseases.

## 2 Results and Discussion

### 2.1 Developing a Hydrogel System with Linear Stiffness Gradient Using GelAGE to Grow MCF7 Breast Cancer Spheroids in Varying Degrees of Volume Restriction in 3D

**Figure 1:**
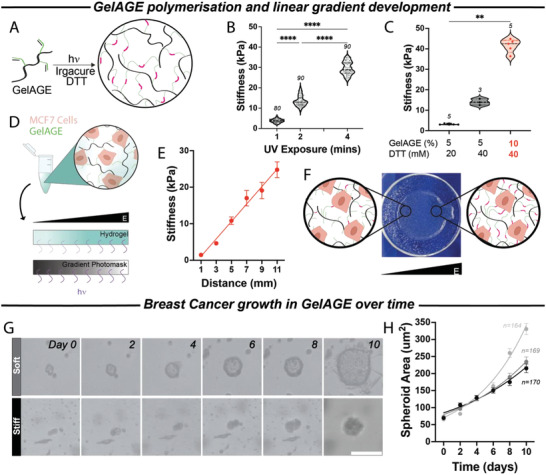
GelAGE is highly tuneable and supports MCF7 spheroid formation. A) synthesis of GelAGE from gelatin by reacting with allyl glycidyl ether (AGE). This attaches allyl functional groups (green) onto the gelatin backbone (black), which form carbon‐carbon double bonds (“enes”) (pink) with each other in the presence of a photoinitiator (Irgacure) and crosslinker (DTT). B) Variable UV exposure to GelAGE (10% GelAGE/0.1% Irgacure/40 mm DTT) showed varying stiffness of the hydrogel from soft (3.8 ± 0.1 kPa), intermediate (13.5 ± 0.3 kPa) or very stiff (29.6 ± 0.4 kPa) (*p* < 0.0001 between all conditions). C) Using constant UV exposure (3 min), the stiffness of hydrogel could be tuned by varying the concentrations of GelAGE and DTT (*p* < 0.01). D) MCF7 cells were added to the precursor GelAGE solution, and a spatial gradient was generated using a gradient transparency photomask. E) After 5.5 min of UV exposure, an ≈1–20 kPa range is established across a 10 mm distance (*n* = 11, *r* ^2^ = 0.74). F) Photo of a polymerized hydrogel at the bottom of a 6‐well glass‐bottom plate. Schematics showing MCF7 cells encapsulated within a gradient stiffness and exposed to varying sized mesh networks. G) Live cell imaging conducted on an Incucyte demonstrated formation of a larger multicellular spheroid from a single MCF7 cell by day 10 in both soft and stiff environment. H) MCF7 spheroid growth displayed exponentially larger areas in softer matrices (*n* = 164, *r* ^2^ = 0.96) than spheroids in the intermediate (*n* = 169, *r* ^2^ = 0.98) and stiff (*n* = 170, *r* ^2^ = 0.98). Numbers above points in graphs (B) and (C) represent the number of samples for each condition. White scale bar represents 100 µm.

Breast tumor growth corresponds to an increase in breast tissue stiffness from 1.1 ± 0.8 to 3.7 ± 1.9 kPa and non‐invasive breast tumors presents a highly heterogeneous stiffness range from ≈1 to 20 kPa, as measured by atomic force microscopy (AFM).^[^ ^12^ ^]^ To mimic the representative stiffness of healthy and cancerous breast tissues, biomaterials such as gelatin with crosslinkable modification, e.g., gelatin methacryloyl (GelMA) have been widely tested.^[^ ^8e^ ^]^ The utilization of thiol‐ene clickable biomaterials such as allylated gelatin (GelAGE, **Figure** **1**A) allowed more precise control over the homogeneity of the hydrogel network during photopolymerization, while exhibiting a similar level of stiffness tuneability to GelMA.^[^ ^9a^ ^]^ The stiffness of GelAGE is highly tuneable, allowing control through either varying photo‐energy levels (e.g., UV exposure time) (Figure 1B) or altered concentrations of GelAGE and DTT (Figure 1C). By using a spatial gradient photomask with 20–100% transparency (Figure 1D), GelAGE hydrogel with a linear stiffness gradient was fabricated with a stiffness range from 1.5 ± 0.5 to 24.8 ± 2.2 kPa across 10 mm length across a circular area (Figure 1E,F). This range of stiffness encompasses the highly heterogeneous range reported for cancerous breast tissue (≈1–20 kPa) measured by the same technique, AFM.^[^ ^12^ ^]^ To study non‐invasive breast tumor growth, MCF7 cells were encapsulated during the photopolymerization of GelAGE (Figure 1D). A glass bottom 6‐well culture plate was used to allow high‐content and high‐resolution imaging after cell encapsulation (Figure 1F). Representative phase contrast images show the growth of a single MCF7 cell into a spheroid over 10 days of culture in both soft and stiff conditions (Figure 1G). The projected area showed a steeper exponential growth trend in soft (3.1 ± 0.5 kPa) regions compared to intermediate (13.9 ± 1.6 kPa) and stiff (21.9 ± 2.2 kPa) regions, indicating varying degrees of restriction and volume expansion in stiffer ranges (Figure 1H). This trend matches the inverse relationship between cell volume and stiffness in a previous experiment using single‐cell culture of adipose‐derived stem cells in GelMA.^[^ ^8e^ ^]^

### 2.2 Volumetric Growth of MCF7 Breast Cancer Spheroids Negatively Correlate to Stiffness of GelAGE Hydrogels

#### 2.2.1 MCF7 Spheroid Growth Displays Matrix Stiffness‐Dependent Volume Expansion across a Gradient in GelAGE Hydrogels

**Figure 2:**
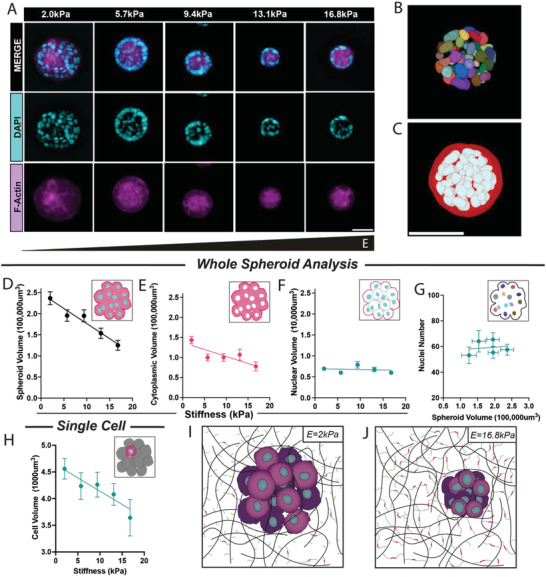
Non‐metastatic MCF7 breast cancer spheroid growth in stiff matrix displays 3D volume restriction. A) Immunocytochemistry was conducted at day 10 so that nuclei (DAPI, cyan) and cell cytoplasm (F‐actin, magenta) could be visualized. These are representative 3D maximum intensity projections from a 30 µm stack from the middle of the spheroid. To gain 3D volumetric analysis, whole raw z‐stack images are processed, segmented and quantified; B) individual nuclei and C) whole spheroid and whole nuclear volume. Images were taken 2 mm apart, spanning 10 mm, with a stiffness ranging from 2.0–16.8 kPa. D) Whole spheroid volume (non‐zero slope, *p* < 0.0001) and E) cytoplasmic volume (non‐zero slope, *p* < 0.0001) F) decreased significantly in stiffer matrix whilst nuclear volume (non‐zero slope, *p* = 0.69) remained unchanged. G) Total nuclei number was compared to spheroid volume and did not display a significant trend (non‐zero slope, *p* = 0.8). H) To confirm changes in whole spheroid volume are driven by cytoplasmic changes, we examined individual cell volume, and as expected in a restricted matrix, cell volume was significantly reduced (non‐zero slope, *p* < 0.05). I,J) Schematic drawings demonstrating the most significant morphological changes between spheroids grown in soft and stiff environments. In each condition 2.0, 5.7, 9.4, 13.1, 16.8 kPa, 53, 50, 44, 51, 44 spheroids were used for the analysis, respectively. White scale bar represents 50 µm.

MCF7 cells were encapsulated at a single cell level and grew into spheroids within GelAGE hydrogels with a linear stiffness gradient. After 10 days, MCF7 spheroids were stained by Rhodamine Phalloidin and DAPI for visualization of actin filaments and nuclei, respectively. Confocal z‐stack images (0.5 µm steps and up to 150 µm thickness) were acquired across the stiffness gradient at 2 mm intervals over 10 mm, at the stiffness of 2.0, 5.7, 9.4, 13.1, and 16.8 kPa. Representative images indicated decreasing sizes of spheroids over increasing stiffness (**Figure** **2**A). For 3D quantification and analysis, a customized FIJI macro enabled the segmentation of whole 3D z‐stack allowing voxel‐based volume calculation of spheroids and nuclei within spheroids (representative 3D reconstruction shown in Figure 2B,C). 3D spheroid data showed an inverse relationship (non‐zero slope, *p* < 0.0001) to stiffness indicating that further volume expansion occurs in a soft (i.e., less restrictive) matrix when compared to a stiffer (i.e., more restrictive) environment (Figure 2D). This inverse relationship matches recent findings of volume expansion of single cells in 3D using elastic (GelMA),^[^ ^8^, ^11^ ^]^ or viscoelastic (alginate) hydrogels for adult stem cell encapsulation.^[^ ^8^, ^13^ ^]^ Pore size and degradability have been intrinsically linked to mechanical confinement in 3D.^[^ ^14^ ^]^ When cells become physically restricted, the confining environment can alter cellular mechanics and morphology, including cell volume, shape and migration. This has been confirmed by a recent study using mouse embryonic fibroblasts, where cell spreading was found to be largely dependent on mesh size.^[^ ^15^ ^]^ Compartmentalizing spheroid volume into cytoplasmic and nuclear volume allowed investigation into the main contributor for spheroid size. Interestingly, as stiffness increased, cytoplasmic volume decreased significantly (Figure 2E, non‐zero slope, *p* = 0.0001) while nuclear volume remained consistent (Figure 2F, non‐zero slope, *p* > 0.05). The average cell volume, i.e., the spheroid volume normalized by nuclear number, was inversely correlated with the stiffness (non‐zero slope, *p* < 0.05) (Figure 2G). Whole spheroid volume did not display a significant correlation with nuclei number (non‐zero slope, *p* > 0.05) (Figure 2F). This data combined confirms that softer matrices (with less crosslinking) produce MCF7 spheroids of larger volume due to the increase of individual cell volume (and expansion) rather than the increase in cell number (depicted in Figure 2I,J).

#### 2.2.2 Soft and Less Restrictive Matrix Shows Higher Levels of Proteins Linking to Volume Expansion, Growth, and Adhesion

**Figure 3:**
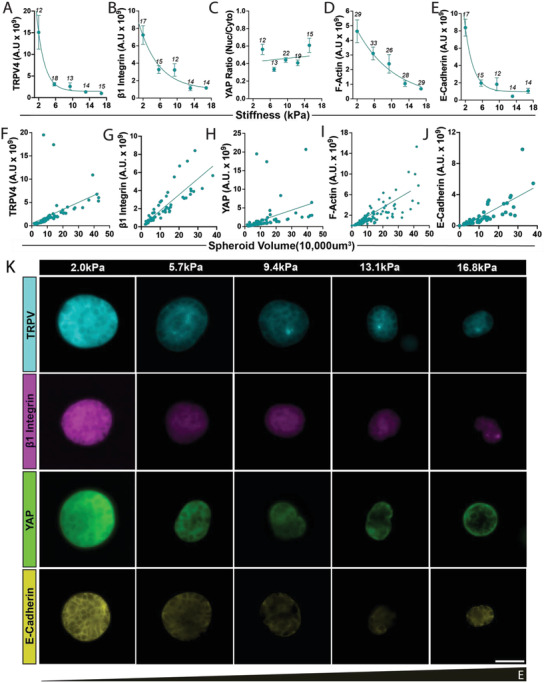
Expression of relevant proteins (TRPV4, β1 integrin, YAP, F‐actin, and E‐cadherin) peak in MCF7 spheroids which experience volume expansion in soft matrix. A–E) Relevant protein expression is significantly higher in 2.0 kPa when compared to most stiffer counterparts (*p* < 0.05) except YAP ratio. F–J) Relevant protein expressions show positive trends with spheroid volume (non‐zero slope, p < 0.001). K) These are representative 3D maximum intensity projections from a 30 µm stack from the middle of the spheroid, serving as a visual representation of protein expression across linear gradient stiffness 2.0–16.8 kPa. Numbers above points in graph (A–E) represent the graded number of spheroids for that condition, (F–J) is the pooled data set for each protein. White scale bar represents 50 µm.

We next wanted to determine whether relevant protein expression (TRPV4, YAP, E‐cadherin, β1 integrin, F‐actin, and Lamin A/C) is relative to volumetric expansion in relation to the surrounding matrix mechanics (**Figure** **3**A–J). Protein expression levels were initially plotted against increasing stiffness and then graphed against relative MCF7 spheroid volume, as volume showed a clear inverse relationship to matrix stiffness (Figure 2D). This enabled us to link protein expression levels not only to stiffness but also to spheroid volume, as the volume in 3D has been reported to regulate cell characteristics, including stiffness, morphology and differentiation.^[^ ^8^, ^16^ ^]^

Previous studies have demonstrated that transient receptor potential vanilloid‐4 (TRPV4) is a calcium‐permeable ion channel that balances osmolarity and subsequently regulates cell volume.^[^ ^17^ ^]^ TRPV4 channels can be activated by membrane stretching^[^ ^18^ ^]^ or forces applied by integrin β1.^[^ ^19^ ^]^ TRPV4 showed a negative trend against stiffness and was significantly elevated in MCF7 spheroids grown in 2.0 kPa compared to stiffer counterparts 9.4, 13.1, and 16.8 kPa (*p* < 0.01) (Figure 3A). Integrin β1 also showed an inverse relationship to increasing stiffness (Figure 3B). When plotted against spheroid volume, both TRPV4 and integrin β1 expression exponentially increased in spheroids of greater volume (*r* ^2^ = 0.997 and 0.97, respectively) (Figure 3F,G). This suggests increased activity of cell‐matrix binding (integrin β1) resulting in greater activation of TRPV4 in softer environments thus enabling larger volume expansion of MCF7 spheroids.

Previous studies have established YAP as a key regulator in the Hippo pathway controlling tissue growth through the translocalization of YAP in either the cytoplasm or the nucleus.^[^ ^20^ ^]^ YAP translocation remained predominantly cytoplasmic with no significant changes across the varying stiffnesses (Figure 3C,L). Although correlated with cell spreading in 2D, YAP nuclear translocalization has been shown to be unrelated or suppressed in 3D,^[^ ^21^ ^]^ and recent investigation has shown YAP activation in breast cancer patient samples only after post‐basement membrane invasion.^[^ ^22^ ^]^ YAP activation has recently been correlated with nuclear flattening, mechanical coupling with stress fibers and opening of nuclear pores when cultured in 2D.^[^ ^23^ ^]^ In our 3D MCF7 spheroid model, F‐actin arrangement remained cortical and failed to form robust stress fibers, and this likely led to inadequate induced tension to the nucleus.

Actin has been shown to be highly regulated in breast cancer, with cortical actin bundle organization occurring in non‐invasive epithelial cells. This has been identified as an important characteristic associated with suppressing invasion and promoting tumor growth and organization.^[^ ^24^ ^]^ F‐actin is linked to adherens junctions via α‐catenin on the cytoplasmic domain of an E‐cadherin.^[^ ^25^ ^]^ E‐cadherin is prominently known for forming cell–cell adhesions with high expression in epithelial cells and is well known to regulate epithelial tissue organization.^[^ ^26^ ^]^ Both proteins display significantly increased expression in the softest condition when compared to all other stiffnesses (*p* < 0.05) (Figure 3E,D). When plotted against spheroid volume, E‐cadherin and F‐actin correlated positively with spheroid volume (*r* ^2^ = 0.933 and 0.98, respectively) (Figure 3I,J). This supports the notion that F‐actin and E‐cadherin are important cytoskeletal and junction proteins responsible for cellular organization in non‐metastatic MCF7 cancer growth.

Studies of cell confinement have linked Lamin A/C expression to mechanosensation and migration.^[^ ^27^ ^]^ Lamin A/C is an intermediate filament responsible for maintaining nuclear stability, preventing DNA damage and mechanotransduction. Photocrosslinkable hydrogels (GelAGE and GelMA) are mostly elastic, with crosslinking chemistry that generates non‐degradable micron‐scale pores. This property produces a mechanical constraint for outward cell expansion.^[^ ^8^, ^28^ ^]^ MCF7 spheroids express increasing Lamin A/C following increasing mechanical constraint, except under the extreme level of confinement where Lamin A/C significantly drops (*p* < 0.01) above 15 kPa (Figure S1, Supporting Information). Recent studies have shown Lamin A/C expression to be non‐monotonic and have a bell‐curve relationship. Increasing confinement leads to increased expression and serves as a mechanism for cells to preserve DNA.^[^ ^27a^ ^]^ However, under excess confinement expression will decrease in favor of a deformable nucleus for invasion.^[^ ^29^ ^]^ This relationship is often seen in cancer, as cells will rupture their nucleus when invading micro‐channels, leading to increased DNA mutation, which is advantageous in tumor progression.^[^ ^27^, ^29^ ^]^

#### 2.2.3 MCF7 Spheroid Stiffness Is Regulated by Changes in ECM Stiffness in 3D

**Figure 4:**
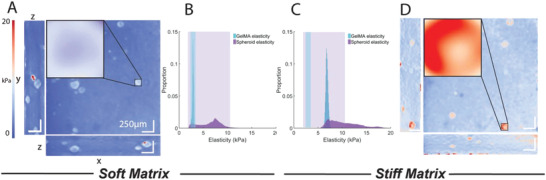
3D mechanical characterization of MCF7 spheroids cultured in soft and stiff GelMA matrix. The mechanical stiffness of A) soft and D) stiff hydrogels with encapsulated spheroids were characterized using QME (stiffness spectrum from 0 kPa, blue to 20 kPa, red). B) GelMA peak (2.9 ± 0.2 kPa) and spheroid peak stiffness (6.4 ± 2.2 kPa) was significantly softer than their stiff counterpart C) GelMA peak (6.8 ± 0.2 kPa) and spheroid peak (10.4 ± 3.1 kPa). White scale bar represents 250 µm.

We use quantitative micro‐elastography (QME) to image spheroid elasticity in 3D and confirm that MCF7 spheroids experience a stiffer surrounding environment with a greater degree of crosslinking. QME is a variant of optical coherence elastography (OCE) that maps 3D micro‐scale elasticity into an image, termed an elastogram. This method enables 3D investigations of elasticity and stiffness and facilitates the non‐invasive mechanical characterization of encapsulated spheroids (**Figure** **4**A,C). In QME experiments, GelMA was used instead of GelAGE due to its higher optical scattering to increase the OCT signal‐to‐noise ratio and improve QME image quality. To further understand increasing Lamin A/C expression from 2.0 to 13.1 kPa range, spheroids were cultured in either soft (1.6 **±** 0.3 kPa) or stiff (12.3 **±** 0.3 kPa) GelMA for 10 days (Figure S2, Supporting Information). QME demonstrates an increased intra‐spheroidal elasticity when cultured in a stiffer environment (10.4 ± 3.1 kPa vs 6.4 ± 2.2 kPa). This suggests increased stiffness, and consequently increased matrix compression, may lead to increased cell stiffness in 3D (Figure 4B,D). With inverse relationships between stiffness and cytoskeletal protein expression (F‐actin and E‐cadherin, Figure 3D,E) in a 3D spheroid model, MCF7 spheroids may utilize increased nuclear lamina (Lamin A/C) expression to reinforce cell structure. This may help regulate the unchanged nuclear volume in a stiffer matrix and support the larger nuc/cyto volume ratio. Many studies have shown that tumor cells will display heterogeneous phenotypes dependent on their spatial location within the tumor.^[^ ^12^ ^]^ This aligns with our QME results which depict peripheral MCF7 cells in contact with ECM at a higher elasticity (Figure 4D).

### 2.3 Decoupling Stiffness of Matrix and Volume of MCF7 Spheroid to Confirm Volume as a Regulator of Relevant Protein Expression

**Figure 5:**
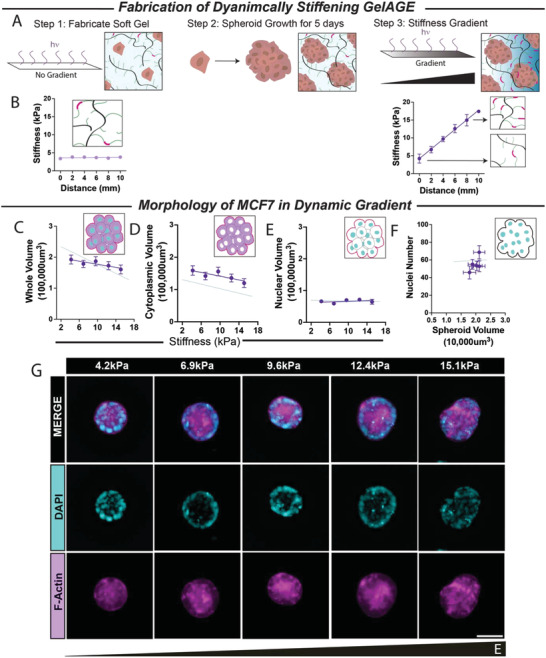
On‐demand stiffening of GelAGE enables equal volume expansion of MCF7 spheroids prior to varying stiffness mechanosensation. A) MCF7 cells are added to GelAGE precursor solution prior to 1st UV polymerization that utilizes a clear photomask for uniform soft matrix and less crosslinking; B) inlet demonstrates many allyl groups not crosslinked (green) versus crosslinked (pink). MCF7 cells will grow into spheroids and experience equal volume expansion in homogenously loose network. After 5 days of growth, the gel undergoes a 2nd UV polymerization with a 20–100% gradient photomask to induce a spatial stiffness gradient with variable crosslinking. This means the degree of crosslinking (pink) increases spatially across the gel. AFM indentations demonstrate uniform 3.8 **±** 0.1 kPa soft matrix at day 0 (light purple, non‐zero slope, *p* > 0.05) and after 5 days a stiffness gradient ranging 4.2–15.1 kPa (dark purple, non‐zero slope, *p* < 0.0001) (B). C) Whole spheroid volume did not display any significant change across different stiffness conditions (*p* > 0.05) nor display a significant trend (non‐zero slope, *p* >0.05). As expected, following no trend in whole spheroid volume, there were no significant trend in D) cytoplasmic volume (non‐zero slope, *p* > 0.05) or E) nuclei volume (non‐zero slope, *p* > 0.05). F) These are representative 3D maximum intensity projections from a 30 µm stack from the middle of the spheroid showing immunofluorescence of nuclei (cyan) stained with DAPI and F‐actin (magenta). In each condition 4.2, 6.9, 9.6, 12.4, 15.1 kPa, there were 53, 50, 44, 51, 44 graded spheroids, respectively. White scale bar represents 50 µm.

In our first platform of a linear stiffness gradient GelAGE hydrogel, the volume of spheroids and matrix stiffness were intrinsically coupled, i.e., soft regions with loose networks allowed larger spheroid volume expansion. For this reason, we developed an on‐demand stiffening platform to allow equal volume expansion with varying stiffness mechanosensation (**Figure** **5**A). MCF7 cells were cultured in a uniformly soft hydrogel (3.8 **±** 0.1 kPa) for 5 days, followed by further photopolymerization of GelAGE to produce a linear stiffness gradient (4.2–15.1 kPa) for the remaining 5 days (Figure 5A,B). A live/dead viability/cytotoxicity assay was conducted after this two‐polymerization step which validated the viability of MCF7 spheroids with exposure to 5.5 min of 2.7 mW cm^−2^ UV with no photomask (Figure S3, Supporting Information). These spheroids will experience less confinement from the surrounding matrix and less 3D volume restriction compared to spheroids grown from single cells. Under reduced confinement, spheroids showed no significant trend in whole spheroid volume (non‐zero slope, *p* > 0.05) (Figure 5C), cytoplasmic volume or nuclear volume (non‐zero slope, *p* > 0.05) (Figure 5D,E). As expected, the average nuclei number per spheroid volume was consistent (non‐zero slope, *p* < 0.05) (Figure 3F). One limitation we observed was that the softest region of this dynamically stiffened GelAGE is already stiffer than the first two soft regions of the previous platform due to the double polymerization. Inadvertently, this resulted in the mean spheroid volume in the softest portion (192 651 **±** 14 365 µm^3^) being equivalent to the volume of the spheroids in the first platform at 5.7 kPa (195 544 **±** 13 621 µm^3^) range, which is smaller than the average volume of spheroids in stiffness of 2.0 kPa (236 648 **±** 15,272 µm^3^).

**Figure 6:**
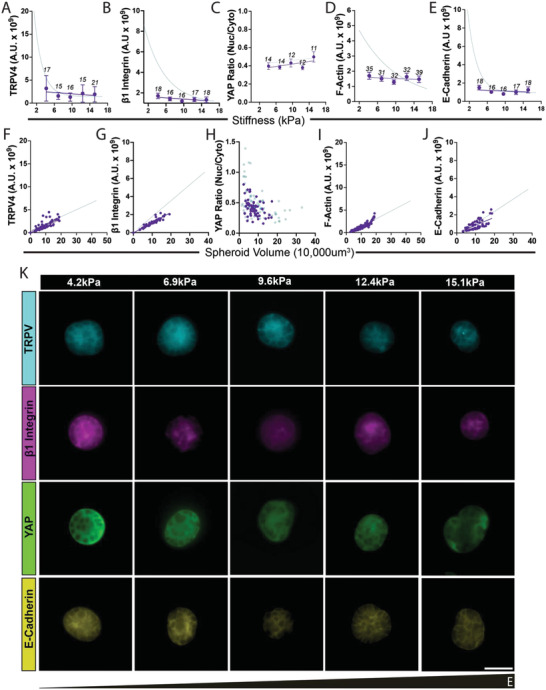
MCF7 spheroids expressed reduced levels of relevant proteins (TRPV, β1 integrin, F‐actin, and E‐cadherin) (dark purple) compared to spheroids of the same size in the previous platform (blue line). Proteins of interest are plotted against either A–E) stiffness or F–J) spheroid volume. G) Representative 3D maximum intensity projections from a 30 µm stack from the middle of the spheroid showing immunofluroescence of relevant proteins. Numbers above points in (A–E) represent number of spheroids sampled and (F–J) represent pooled data. White scale bar represents 50 µm.

When we investigated relevant protein expression (TRPV4, integrin β1, YAP, F‐actin and E‐cadherin) in MCF7 spheroids of equal volume expansion platform, all proteins displayed no change to expression across the stiffness gradient (**Figure** **6**A–E). However, when we compare the expression of these proteins to spheroid volume in both platforms it becomes evident that volume is still responsible for relevant (TRPV4, integrin β1, F‐actin, E‐cadherin) protein expression (Figure 6F–J).

## 3 Conclusion

In diseased models, the extracellular matrix develops into a highly dynamic niche. In order to maintain physiological relevance, biomaterials must offer a higher degree of tuneability. GelAGE hydrogels are readily tunable providing variable stiffness through manipulation of constituent concentrations or photoenergy (e.g., UV) exposure. Establishing a linear gradient stiffness GelAGE hydrogel enabled high throughput analysis of spheroid morphology through a range of 1–20 kPa, encompassing the range previously reported for healthy and diseased breast tissue. Due to the coupling between stiffness and mesh size (network), increasing stiffness will result in increased mechanical confinement of encapsulated cells. Instead of chemically inducing hypo‐osmotic and hyper‐osmotic conditions to investigate volume‐regulated spheroid morphology, GelAGE hydrogels can be utilized to mechanically induce volume expansion via on‐demand stiffening. Utilizing two‐polymerization steps allows for equal volume expansion of spheroids prior to stiffness affliction. Overall, this biomaterial enabled us to confirm that relevant protein expression (TRPV4, integrin β1, F‐actin, and E‐cadherin) is regulated by volume expansion rather than stiffness cues and 2D mechanomarkers, YAP and Lamin A/C, showed less clear trend in 3D environment.

## 4 Experimental Section

### Molds, Coverslip, and Well‐Plate Preparation

Molds were designed on Fusion 360 (AutoDesk), printed with clear resin (V4, Formlabs) using a 3D printer (Form 2; Formlabs), washed with isopropyl alcohol (IPA) for 10 min and cured for 20 min (Formlabs) using 405 nm UV light at 60 °C. Sandpaper was used to even the surface of the mold. Prior to polymerization, 2 drops of dichlorordimethylsilane (DCDMS; Sigma) were added per mold, and either side of each 15 mm diameter circular coverslip (Menzel‐Gläser) to prevent GelAGE sticking. 100 µL of a solution containing 20 mL of 100% ethanol, 600 µL acetic acid (3% v/v) and 3‐(trimethoxysilyl)propyl methacrylate (0.5% v/v) (Merk) was added to each well in a glass bottom, 6‐well plate (20 mm micro‐well, #1.5 coverglass; Cellvis) for 5 min. The well‐plate was then left to dry in the fume hood, at room temperature.

### Cell Culture

MCF7 (non‐metastatic breast cancer) cells were cultured using T‐75 flasks (Conoco) inside an incubator (37 °C with 5% CO2) until 80% confluency was reached. The media consisted of high glucose Dulbecco's Modified Eagle Medium (hg‐DMEM; Gibco), with 1% (v/v) antibiotic‐antimycotic (anti‐anti; Gibco) and 10% (v/v) foetal bovine serum (FBS; Gibco). Media was changed every 2 days. To passage MCF7 cells, 2 mL of 0.25% Trypsin‐ethylnediaminetetraacetic acid solution (Sigma) (w/v) was used to facilitate cell retrieval. Once cell detachment and rounding were observed, 5 mL of Hg‐DMEM was added and pipetted over the culture surface to ensure maximum cell recovery. Cells were then transferred into a 15 mL tube and centrifuged at 1200 rpm for 5 min. The supernatant was discarded, and the cell pellet was resuspended in 1 mL of Hg‐DMEM. Cells were then populated using a hemocytometer and Trypan Blue (Sigma).

### GelAGE Fabrication

Allyl‐functionalised gelatin (Gel‐AGE) was synthesized according to previously published protocol.^[^ ^9^, ^30^ ^]^ Gelatin (10 wt%) was firstly dissolved in deionized water at 50 °C, followed by addition of 2.4 mmol of AGE and 0.4 mmol of NaOH per 1 g of gelatin, relatively. The reaction was carried out at 65 °C for 8 h, then diluted 10× in deionized water, followed by dialysis against deionized water (MWCO: 12–14 kDa) for 3 days at room temperature. After dialysis, the final product was lyophilised to obtain dried Gel‐AGE macromer. GelAGE solution was processed through a desiccator vacuum for 15 min prior to being dissolved in 1x phosphate buffer (PBS; pH7.4, Gibco) to create 10% (w/v) concentration. This solution was dissolved in a water bath (at 50 °C) for 25 min, also reducing viscosity. The crosslinker Dithiothreitol (DTT; Sigma) was dissolved in PBS achieve a stock concentration of 400 nm (61.7 mg mL^−1^). The light sensitive, photoinitiator, Irgacure (Ig2959; Sigma) 2‐Hydrocy‐4’‐(2‐hydrocyethoxy)‐2‐methylpropiophenone, was dissolved in 100% ethanol to achieve a stock concentration of 10% (v/v). Both were added in the desired final concentration to the GelAGE solution; for this experiment it was 10% GelAGE/20 mm DTT/0.1% Ig2959. The final working solution was kept in a 37 °C water bath and wrapped in aluminium foil until polymerization.

### Linear Gradient GelAGE Fabrication and Cell Encapsulation

For encapsulation of individual cells, MCF7 cells were resuspended in GelAGE precursor solution (1000 MCF7 cells per hydrogel; 7.69 cells µL^−1^). The cell‐laden GelAGE was pipetted in the mold in the glass‐bottom 6‐well plate and covered with a DCDMS coverslip. To establish a linear gradient stiffness a 20–100% transparent acetate photomask was attached to the underside of the well. Cell‐laden GelAGE was exposed to UV light (360 nm, 3.6 mW cm^−2^) for 5.5 min before mold and coverslip were removed, and growth media added. Routine cell culture took place for 10 days, before fixation and staining.

### On Demand Linear Gradient GelAGE Fabrication

For cells to experience equal growth expansion prior to gradient stiffness, gels must undergo a two‐stage polymerisation. The cell‐laden GelAGE solution was pipetted into the same construct as previous experiment, except a 100% transparent photomask was adhered to the underside of glass‐bottom well plate and cell‐laden gels were exposed to 1.5 min of UV light (360 nm, 3.6 mW cm^−2^). After 5 days of culture, Ig2959 was added to growth media to end concentration of 0.1% for 30 min. This solution was removed, the mold and 200 µL of 0.1% of Ig2959 diluted in 100% ethanol was added per gel and covered with DCDMS coverslip. A 0–100% transparent acetate photomask was added to the underside of each well, and the gels were exposed to UV light for 4 min. Mold and coverslip were removed, and growth media added. Routine cell culture was performed for the remaining 5 days of culture, before fixation and staining protocol.

### Live Cell Imaging of Spheroid Growth in Three‐Dimensional Hydrogels

6 well glass‐bottom plates with polymerized cell‐laden GelAGE were placed into the IncyCyte (Sartorius). Using multi‐spheroid function and the capture set to brightfield and phase contrast, images were taken every 6 h. Live‐cell time‐lapse imaging was performed over the entire 10‐day culture period.

### Atomic Force Microscopy

The stiffness of the hydrogels was determined via surface indentation using an MFP‐3D Origin atomic force microscope (AFM; Asylum Research). Triple indentations were performed at 2 mm intervals along the gradient using a 200 µm gold‐coated, silicon‐nitride pyramid‐shaped cantilever tip (PRP‐TR‐20; NanoWorld). The tip approached at 2 µm s^−1^ until a 2 nN trigger force was registered and then retracted at 10 µm s^−1^. The stiffness was characterised using the contact‐generated force graphs and Igor Pro posthoc analysis.^[^ ^31^ ^]^ An average from each triplet indentation was calculated. This process was repeated across each batch of fabricated GelAGE to ensure consistency.

### Immunocytochemistry

Fixation and Immunofluorescence were performed on day 10 of spheroid culture. Gels were transferred to a fume hood and washed three times with PBS and fixed with 4% (w/v) paraformaldehyde (PFA; Chem‐Cruz) for 30 min at room temperature. Between each step gels were washed three times with PBS (2 min each time). Gels were permeabilized with 1% (w/v) Triton‐X‐100 (Sigma) for 30 min. Blocking was done with 5% (v/v) goat serum for 1 h at room temperature. The following primary antibodies were used; YAP (mouse, sc101199; Santa Cruz Biotechnology), Lamin‐A/C (mouse, sc7292; Santa Cruz Biotechnology), E‐cadherin (rabbit, 24E10; Cell Signaling Technology), TRPV4 (rabbit, ab39260; Abcam), β1 integrin (mouse, ab3094; Abcam). All were used as a 1:100 dilution in 2% bovine serum albumin (BSA; Sigma) in PBS. These were incubated at 37 °C for 2 days. The respective secondary antibodies (all 1:200) were added; Alexa Fluor 488 goat anti‐mouse (a11001; Abcam), Alexa Fluor 488 goat anti‐rabbit (a11008; Abcam), and Alexa Fluor 594 goat anti‐rabbit (ab150080; Abcam) and Alexa Fluor 594 goat anti‐mouse (ab150116; Abcam). Phalloidin‐iFluor 647 (ab176759; Abcam) was added at a 1:1000 dilution. Secondary antibody and phalloidin dilutions were in BSA, and gels were incubated for 2 h at 37 °C. 1:400 DAPI (D9542; Sigma) in PBS was added to the gels for 40 min at room temperature and washed for 30 min in PBS before being mounted with SlowFade Diamond Mounting Agent (Life Technologies) and stored at 4 °C until imaged with Delta Vision.

### Viability Assay

Utilising the same protocol as On Demand Linear Gradient Hydrogel Fabrication, spheroids were cultured for 5 days before utilising the LIVE/DEAD Viability/Cytotoxicity kit, for mammalian cells (L3224, Sigma). Hydrogels were washed 3 times with PBS for 5 min before addition of solution containing; 4 µm Calcein AM and 8 µm Ethidium homodimer‐1 diluted in PBS. This was left to incubate for 40 min before capturing on Delta Vision.

### Delta Vision Microscopy

All stained hydrogels are imaged by Delta Vision Elite (GE) controlled by softWoRx software. A 40× oil immersion lens was used to capture Z‐series of images at a resolution of 1024 × 1024 pixels and 0.5 µm steps. Spheres were imaged every 2 mm over a 10 mm span along the stiffness gradient, corresponding with AFM indentations. All captures underwent post‐processing functions including deconvolution and saved as dv. files for data analysis.

### Quantitative Micro‐Elastography

Optical coherence elastography (OCE)has been used to map spheroid and ECM elasticity in 3‐D. Specifically, QME is used, a variant of compression based OCE.^[^ ^32^ ^]^ QME has been demonstrated imaging the elasticity of individual cells in 3‐D,^[^ ^8g^ ^]^ and more recently, a high‐resolution variant of QME, known as mechano‐microscopy, has been demonstrated imaging the elasticity of sub‐cellular features in 3‐D.^[^ ^33^ ^]^ In QME, the sample is brought into contact with a rigid glass imaging window and rigid plate. The rigid plate is mounted on a motorized axial translation stage, which is used to apply a preload strain of ≈5% to the sample to ensure uniform contact between the imaging window, sample, and rigid plate. An additional microscale compressive loading is then applied using a ring actuator attached to the rigid plate. Optical coherence tomography (OCT) is used to acquire volumetric images of the sample before and after the microscale compression by synchronizing the actuator loading with the OCT scan acquisition. The OCT system illuminates the sample through the glass imaging window using a focused beam of nonionizing, near‐infrared light. Here, a fiber‐based spectral‐domain OCT system (Telesto 220, Thorlabs Inc., USA) that has a spectral bandwidth of 170 nm and a central wavelength of 1300 nm was used. The experimentally measured axial and lateral OCT resolutions (full‐width at half maximum of irradiance) are 4.8 and 4.4 µm, respectively. The OCT system was operated in a dual‐arm configuration and scans were acquired by acquiring 1000 A‐scans per B‐scan, and 2000 B‐scans per C‐scan over a 2.2 mm × 2.2 mm lateral region.^[^ ^34^ ^]^ Assuming a sample refractive index of 1.4, the resulting OCT voxel size was 2.2 µm × 2.2 µm × 2.4 µm (*xyz*). The ring actuator was driven in a quasi‐static regime by a 10 Hz square wave collinearly with the OCT imaging beam and synchronized with the acquisition of OCT B‐scans. Two B‐scans were acquired for each *y*‐location such that alternate B‐scans are acquired at different compression levels. Local axial displacement in the sample resulting from the microscale compression is calculated from the OCT phase difference between B‐scans acquired at the same *y*‐location.^[^ ^35^ ^]^ Local axial strain is calculated from the gradient of axial displacement with depth using weighted‐least squares linear regression. Spatial averaging is used to alleviate the impact of noise in the displacement measurements on strain sensitivity where the resulting strain spatial resolution is ≈35 µm (isotropic).^[^ ^36^ ^]^ To measure local axial stress in the sample, a compliant silicone layer is placed between the sample and rigid plate. In previous studies, the compliant layer is typically placed between the imaging window and the sample.^[^ ^37^ ^]^ However, in this study, as the samples were thin (<1 mm) and had relatively low optical scattering with sparsely distributed spheroids, it was possible to image all the way through the samples. Therefore, the compliant layer was placed between the sample and rigid plate, as described previously.^[^ ^33^ ^]^ This configuration removes attenuation of the beam from passing through the compliant layer before reaching the sample, maximizing the OCT signal‐to‐noise ratio (SNR) in the spheroids. The thickness of the compliant layer is measured before and after the preload strain is applied to the sample to obtain an estimate of the preload strain at each point on the sample's surface. The local axial strain measured in the layer is used to calculate the local axial stress at the silicone layer–sample interface. The stress‐strain relationship of the compliant layer is characterized using a uniaxial compression testing apparatus as described previously.^[^ ^32^ ^]^ Under the assumption that stress is uniaxial, and by knowing both the local axial stress and local axial strain, the sample's local elasticity is calculated as a tangent modulus at the point of preload strain. At low preload strains, tangent modulus is equivalent to Young's modulus. The spatial resolution of elasticity is ≈35 µm (isotropic) with a typical scan acquisition time of less than 2 min. In QME experiments, GelMA was used instead of GelAGE due to its higher optical scattering to increase the OCT SNR and improve QME image quality.

### GelMA Fabrication

GelMA polymer was produced based on previously described method.^[^ ^8e,f^ ^]^ Precursor solution was made by dissolving lyophilized GelMA in PBS with Ig2959 (diluted to 10% concentration in 100% ethanol) to an ending concentration of 10% GelMA/0.1% Ig2959. The same protocol for linear gradient gel fabrication was used.

### Image Analysis

Image analysis was scripted in ImageJ/FIJI with additional processing and analysis plugins installed.^[^ ^38^ ^]^ Images were generated and processed with the same settings and equipment to ensure standardization. Spheroids were processed as previously described.^[^ ^11b^ ^]^ In addition to this, a Gaussian weighted median (Biovoxxel) filter was applied to the DAPI channel prior to nuclei segmentation. While F‐actin was used for whole spheroid gating, both channels underwent pre‐processing and segmentation (marker‐controlled watershed) which was accelerated with CLIJ2 and analysed with Morpholibj.

### Statistical Analysis

All statistical analysis was performed in GraphPad PRISM. A normal gaussian distribution was used to test for normality and lognormality. For non‐normal distribution or normal distribution, a Kruskal–Wallis Test or one‐way ANOVA was used to detect significant differences, respectively. Unless stated otherwise it can be assumed a Kruskal–Wallis test was used to detect significance. A minimum of three biological replicated were analysed for each experiment.

## Conflict of Interest

The authors declare no conflict of interest.

## Supporting information

Supporting Information

## Data Availability

The data that support the findings of this study are available in the supporting information of this article.
