# Supplementary material for: 3D Volumetric Mechanosensation of MCF7 Breast Cancer Spheroids in a Linear Stiffness Gradient GelAGE
Source: Adv Healthc Mater. 2023 Sep 19;12(31):2301506. doi: 10.1002/adhm.202301506 (PMC11481087; doi:10.1002/adhm.202301506)
Supplement: Supplementary file 1 — Supporting Information [file ADHM-12-2301506-s001.pdf]

ADVANCED HEALTHCARE MATERIALS

Supporting Information

for *Adv. Healthcare Mater.*, DOI 10.1002/adhm.202301506

3D Volumetric Mechanosensation of MCF7 Breast Cancer Spheroids in a Linear Stiffness Gradient GelAGE

*Danielle Vahala, Sebastian E. Amos, Marta Sacchi, Bram G. Soliman, Matt S. Hepburn, Alireza Mowla, Jiayue Li, Ji Hoon Jeong, Chrissie Astell, Yongsung Hwang, Brendan F. Kennedy, Khoon S. Lim and Yu Suk Choi**

Supporting Information

3D Volumetric Mechanosensation of Breast Cancer Spheroids in a Linear Stiffness Gradient GelAGE

*Danielle Vahala, Sebastian E. Amos, Marta Sacchi, Bram G. Soliman, Matt S. Hepburn, Alireza Mowla, Jiayue Li, Ji Hoon Jeong, Chrissie Astell, Yongsung Hwang, Brendan F. Kennedy, Khoon S. Lim, Yu Suk Choi**

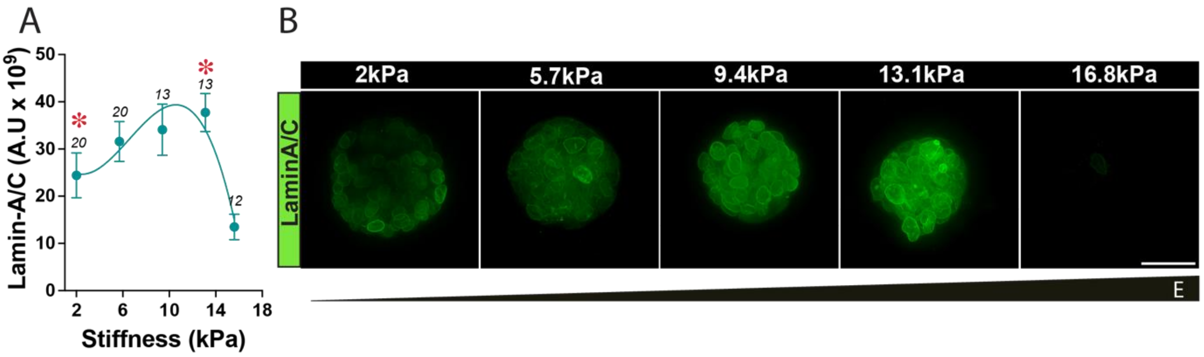

Supplementary Figure 1. Lamin-A expression displays a non-monotonic relationship with increasing mechanical stimuli. A) Lamin-A intensity scales with increasing stiffness, up until extreme confinement (13.1 kPa) after which expression significantly drops when compared to softer conditions, excluding 2.0 kPa ($p<0.05$). (*) references the stiffness points used for OCE measurements. B) Representative fluorescence of Lamin-A across a spanning gradient of 2.0 - 16.8 kPa. Numbers above points in graph A represent graded number of spheroids for that condition, B is the pooled data set.

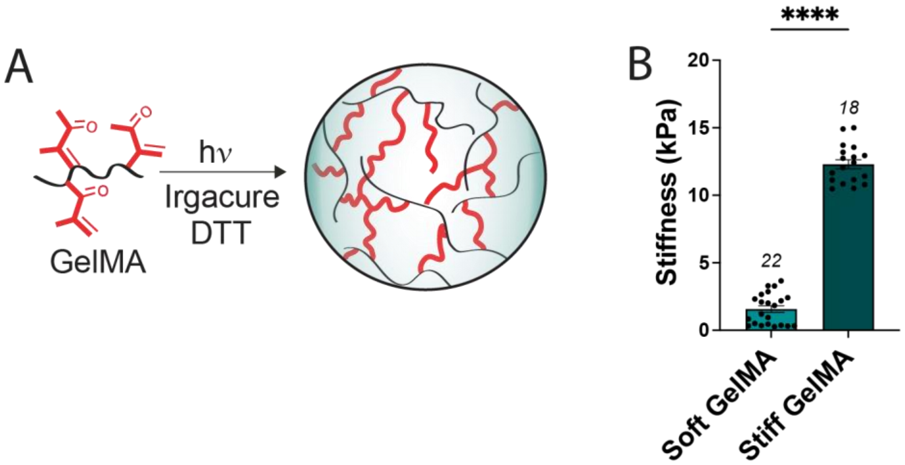

Supplementary Figure 2. GelMA offers similar biomechanical cues as GelAGE. A) Similar to GelAGE they both contain gelatin backbone and undergo free radical polymerization. B)

GelMA with UV exposure for 25 seconds resulted in significantly softer hydrogel (1.6 ± 0.3 kPa) when compared to 50 seconds of exposure (12.3 ± 0.3 kPa) ($p<0.0001$, one-way ANOVA). Numbers above points in graph B represents the number of triplet indentations measured by AFM.

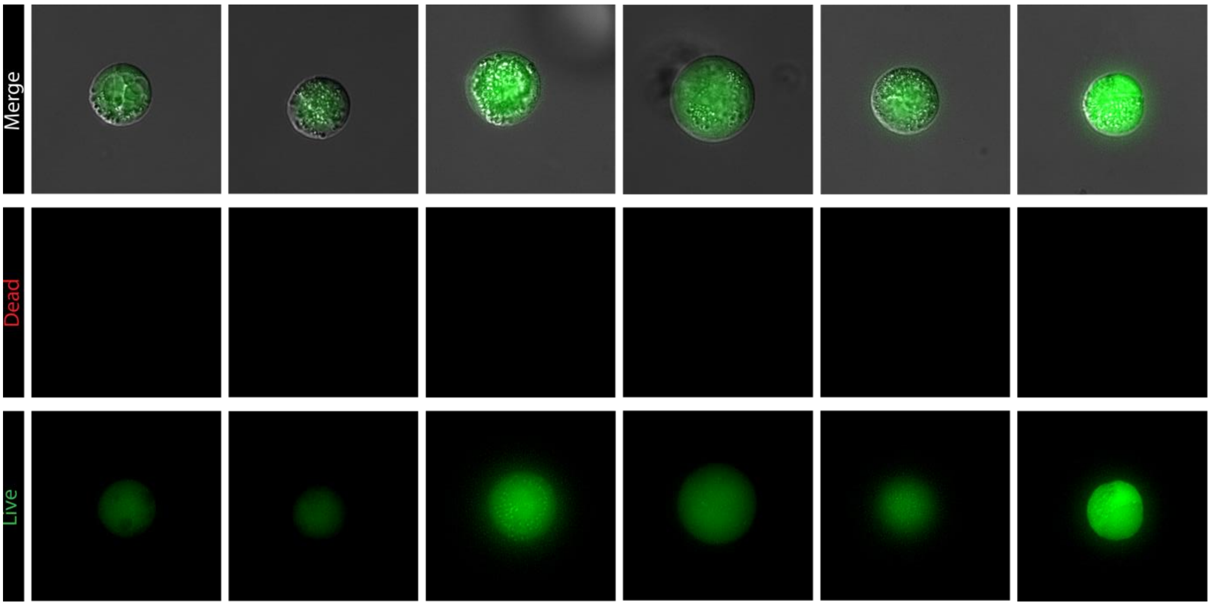

Supplementary Figure 3. Max projections of Live (green)/Dead (red) Viability/Cytotoxicity assay of MCF7 spheroids cultured for 5 days with a total of 5.5 minutes of UV exposure at an average of 2.69 mw/cm^2.
